# Metagenomics analysis of microbial communities associated with a traditional rice wine starter culture (*Xaj*-*pitha*) of Assam, India

**DOI:** 10.1007/s13205-016-0471-1

**Published:** 2016-07-15

**Authors:** Sudipta Sankar Bora, Jyotshna Keot, Saurav Das, Kishore Sarma, Madhumita Barooah

**Affiliations:** Department of Agricultural Biotechnology, Assam Agricultural University, Jorhat, 785013 Assam India

**Keywords:** Metagenomics, Starter culture, Taxonomy, Rice wine and microflora

## Abstract

This is the first report on the microbial diversity of *xaj*-*pitha*, a rice wine fermentation starter culture through a metagenomics approach involving Illumine-based whole genome shotgun (WGS) sequencing method. Metagenomic DNA was extracted from rice wine starter culture concocted by *Ahom* community of Assam and analyzed using a MiSeq^®^ System. A total of 2,78,231 contigs, with an average read length of 640.13 bp, were obtained. Data obtained from the use of several taxonomic profiling tools were compared with previously reported microbial diversity studies through the culture-dependent and culture-independent method. The microbial community revealed the existence of amylase producers, such as *Rhizopus delemar, Mucor circinelloides*, and *Aspergillus* sp. Ethanol producers viz., *Meyerozyma guilliermondii, Wickerhamomyces ciferrii, Saccharomyces cerevisiae, Candida glabrata, Debaryomyces hansenii, Ogataea parapolymorpha*, and *Dekkera bruxellensis*, were found associated with the starter culture along with a diverse range of opportunistic contaminants. The bacterial microflora was dominated by lactic acid bacteria (LAB). The most frequent occurring LAB was *Lactobacillus plantarum, Lactobacillus brevis, Leuconostoc lactis, Weissella cibaria, Lactococcus lactis, Weissella para mesenteroides, Leuconostoc pseudomesenteroides*, etc. Our study provided a comprehensive picture of microbial diversity associated with rice wine fermentation starter and indicated the superiority of metagenomic sequencing over previously used techniques.

## Introduction

The methodology of rice wine preparation is more or less similar among most of the ethnic communities of Assam, a north-eastern state of India; however, fermentation starters (Assamese name *xaj*-*pitha*) and the substrates differ resulting in variance in the quality of the final product (Tanti et al. 2010; Das et al. 2012). The use of fermentation starters is believed to have originated in China from where it spread to the other neighboring countries in Asia (Hanai 1992). Various local names for rice wine starters are used in Asian countries, such as *banh men* in Vietnam, *chu* in China, *koji* in Japan, *nuruk* in Korea, *murcha* in India, *ragi* in Indonesia, *ragi tapai* in Malaysia, and *bubod* in Philippines (Limtong et al. 2005). In Assam, different ethnic communities also have their own terms to refer to such fermentation starter cultures. The starters are mixed cultures of molds, yeasts, and bacteria that are maintained on substrate, such as rice powder, supplemented with various herbs.

The indigenous microbial diversity in various sources of local beverages could be a rich resource for oenological research. Few studies have reported microbial diversity of rice wine fermentation starters (Mao and Odyuo 2007; Xie et al. 2007; Rong et al. 2009; Shi et al. 2009). However, most of these studies examined the microbial community of wine starters using culture-dependent methods that might not have permitted identification of some important microbial species, as their growth requirements are unknown, and hence, a comprehensive picture of the microbial diversity in rice wine starters is yet to be reported (Thanh et al. 2008).

The first culture-independent study of starter microbiota was reported in the traditional Vietnamese alcohol fermentation starters (*banh men*) through Polymerase Chain Reaction (PCR)-based Denaturing Gradient Gel Electrophoresis (DGGE) (Thanh et al. 2008). The study revealed a remarkable diversity of fungal microflora among various samples; however, the bacterial community exhibited a rather “spontaneous” species composition among the collected starters. Although culture-independent approaches, such as Polymerase Chain Reaction (PCR)-based amplification and sequencing of 16S rRNA genes or Denaturing Gradient Gel Electrophoresis (DGGE), have proven to be powerful tools in investigating different types of traditional fermentations (Meroth et al. 2003; Prakitchaiwattana et al. 2004; Rantsiou et al. 2005; Haruta et al. 2006), the technique is unable to reflect the overall microbial diversity due to low throughput (Ercolini 2004; Prakitchaiwattana et al. 2004). On the other hand, high-throughput sequencing (HTS) methods, such as 454 pyrosequencing and Illumina sequencing technologies, have been recently applied as novel promising methods to investigate microbial communities in different habitats. DNA-based high-throughput-sequencing metagenomics have been applied to reveal microbial communities in marine water (Gilbert et al. 2008), soil (Urich et al. 2008), oral cavities (Lazarevic et al. 2009), human guts (Qin et al. 2010), etc. HTS has also been used for exploring the microbial community structure of a variety of fermented foods and beverages; e.g., *Kimchi*, a traditional Korean fermented food (Jung et al. 2011), Irish soft, semi-hard, and hard cheeses (Quigley et al. 2012), Danish raw milk cheeses (Masoud et al. 2011), *Kefir* grains (used in fermentation of *Kefir*, a traditional Turkish drink) (Nalbantoglu et al. 2014), Chinese rice wine (Hong et al. 2016), etc. Moreover, HTS was also instrumental in unveiling the microbial succession of Lactobacillales and yeasts over the members of the Enterobacteriaceae in brewing American coolship ale (Bokulich et al. 2012). Based on the HTS analysis and carbohydrate utilization pattern, *Lactobacillus fabifermentans* was identified to be one of the most dominant bacterial species involved in grape marc fermentation (Campanaro et al. 2014). Based on these observations, it can be concluded that the HTS analysis could provide more insights into the microbial communities and also reflects their role on fermentation processes.

However, studies on microbial community diversity through metagenomics approach in rice wine culture is yet to be reported. Our study is the first of its kind to use the whole genome shotgun (WGS) sequencing to analyze the microbial community occurring in the starter culture traditionally used in Assamese rice wine fermentation. We aimed at identifying the key microbial communities associated with traditional starter culture (*xaj*-*pitha*) and attempted to find relatedness of the microbiota with that of the previously analyzed starters from south-east Asia.

## Materials and methods

The starter is prepared by concoction of rice flour and several herbs. These herbs are believed to impart intoxicating property to the liquor (Sarma 2002). Apart from contributing various organoleptic properties to the wine, these various plants are also said to have many other medicinal properties (Das et al. 2012). Some of the plant extracts may also provide certain nutrients for the survival and growth of the microflora present in the starter cakes (Thanh et al. 2008).

The fermentation in rice wine is known to be a consortia effect of several biochemical and ecological processes, where yeast strains play a major role in ultimate conversion of fermentable sugar to alcohol and esters. Usually, glutinous rice (local name *Bora*) is first steamed and then allowed to cool on a bamboo mat. Powdered starter (four starter cultures per kg of rice) is sprinkled on the cooked rice and mixed thoroughly. This mixture is packed into an earthen pot, properly sealed with fresh leaves of *Dryopteris* sp. (local name *dhekia*). Several of these pots are kept atop of a top bench (local name *dhowa*-*chang*) over fire place. Incubation results in the production of a light-yellow alcoholic beverage, called as *rohi*, are collected from the pot. *Rohi* has a sweet taste and a strong aroma. It is decanted and diluted with water to serve as *xaj.*

### Sampling

Five rice wine starter samples belonging to rural households were collected from the Titabar sub-division (26.60°N, 94.20°E) of Jorhat district (state of Assam, India) on August 20, 2014. All the samples were collected from people belonging to Ahom community of the study area. The Ahoms of the Tai-Shan family came from Burma across the Patkai range and entered Assam under an adventurous leader Sukapha. The special section to which they belonged, or the Shans proper, occupied the northern and eastern hill tracts of Upper Burma and Western Yunnan, where they formed a group of states for which, according to Ney Elias, there is no collective native name. The Ahoms subdued the various local chiefs through a series of determined and skilful moves, and very soon firmly entrenched themselves as the masters over a long tract. Skilled women from rural background of this community are engaged in the traditional *Xaj* preparation.

The collected samples were transported to the laboratory on ice packs and were immediately subjected for the proximate analysis using the standard procedure. Moisture, crude fat, and ash contents of collected starter samples were determined through the standard methods (AOAC 1970). The total nitrogen content of fat-free samples was estimated by the Micro-Kjeldahl method, and crude protein was calculated by multiplying the total *N* by 6.25 (Balasubramaniam and Sadasivam 1987). Crude fiber was determined using the method described by Sadasivam and Manickam (1996). Total soluble sugar was determined using the Anthrone method (Yemm and Willis 1954). The reducing sugar was estimated by the standard biochemical method using 3,5-di-nitrosalicylic acid (DNS) reagent with slight modifications (Somogyi 1952). The non-reducing sugar content was derived by subtracting the percentage of reducing sugar from the percentage of total soluble sugar. Results of biochemical composition of the samples have been presented in Table 1. One gram of starter was weighed from each powdered sample and then pooled as a composite sample in sterile laboratory conditions.

**Table 1:** Biochemical composition of starter cultures collected from Titabar sub-division, Jorhat district, Assam, India

Sample code	Weight in gm/shape	Moisture	Crude fat %	Crude protein %	Crude fiber %	Ash %	Starch %	Total soluble sugar %	Reducing sugar %	Non-reducing sugar %
ABT-S4J3	11.598/round	13.61	0.76	7.48	1.863	1.13	74.58	1.211	0.297	0.914
ABT-S5J3	12.024/oval	14.46	0.96	8.74	2.006	1.27	72.39	1.267	0.276	0.991
ABT-S6J3	12.560/oval	14.34	0.87	8.19	1.762	1.14	73.37	1.013	0.345	0.668
ABT-S7J3	14.462/round	13.89	0.86	7.36	1.634	0.83	75.29	1.027	0.297	0.730
ABT-S8J3	12.220/oval	13.21	0.76	6.38	2.224	0.61	76.78	1.164	0.067	1.097

### DNA isolation from starter samples

DNA was isolated from 1 g of composite starter sample using Environmental gDNA isolation kit (Xcelgen, India). Extracted DNA was quantified using Qubit fluorometer according to manufacturer’s instructions.

### Preparation of 2 × 300 MiSeq library

The paired-end sequencing library was prepared using illumina TruSeq DNA Library Preparation Kit, initiated with fragmentation of 1 µg gDNA, followed by paired-end adapter ligation. The ligated product was purified using 1X Ampure beads. The purified product was subjected to size-selection at ~500–800 bp, and the selected product was PCR amplified as described in the kit protocol. The amplified library was analyzed in Bioanalyzer 2100 (Agilent Technologies) using high-sensitivity (HS) DNA chip as per manufacturer’s instructions.

### Cluster generation and sequencing

**Fig. 1:**
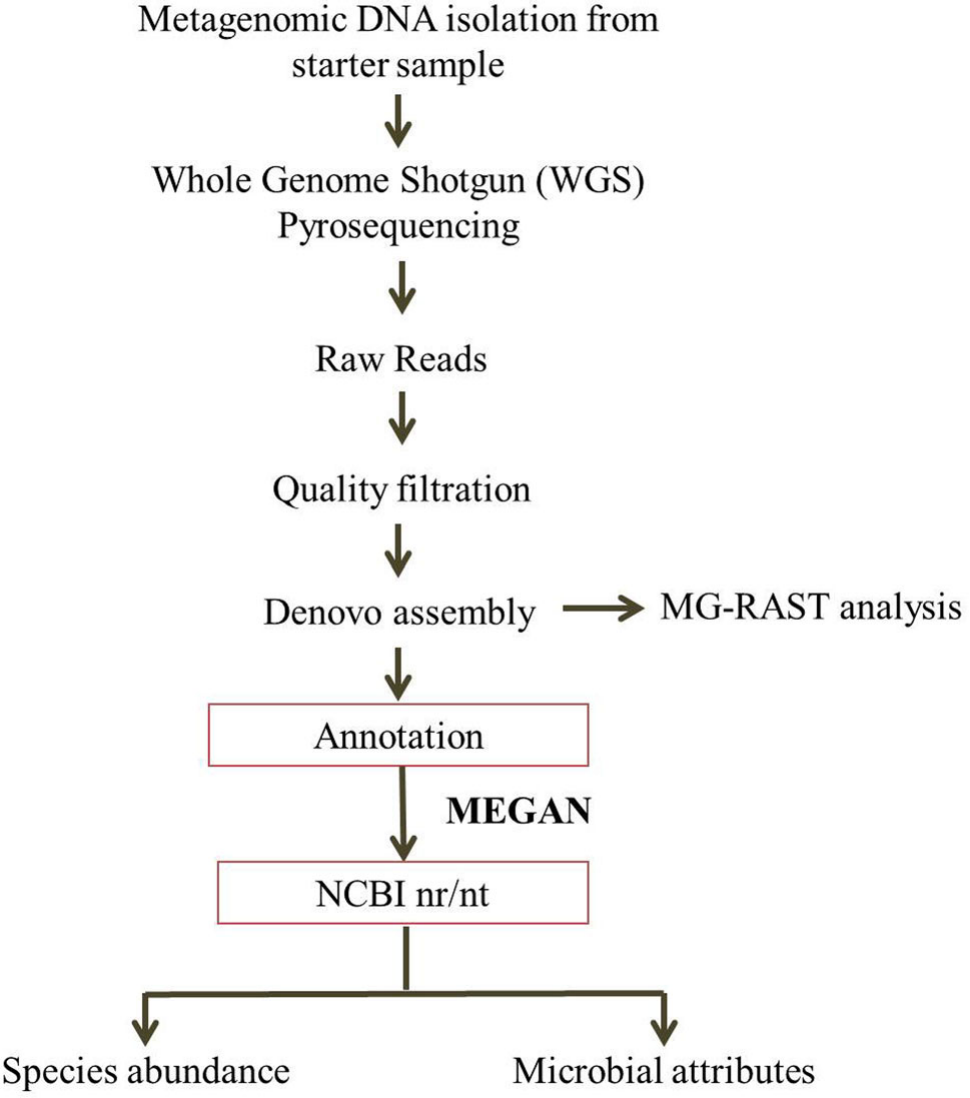
Analysis strategy performed to analyze microbial diversity prevalent in the starter culture sample. DNA from an efficient starter sample was used for whole genome shotgun (WGS) sequencing

After obtaining the Qubit concentration for the library and the mean peak size from Bioanalyzer profile, 10 pM of library was loaded onto Illumina MiSeq for cluster generation and sequencing. Paired-end sequencing allows the template fragments to be sequenced in both the forward and reverse directions on MiSeq. The reagents supplied with the kit were used in the binding of samples to complementary adapter oligos on paired-on flow cell. The adapters were designed to allow selective cleavage of the forward strands after resynthesis of the reverse strand during sequencing. The copied reverse strand was then used to sequence from the opposite end of the fragment. High-quality metagenome reads were assembled using CLC workbench (CLC bio, Denmark) at default parameter (minimum contig length 200) for trimming and *de novo* assembly (Chan et al. 2012). In Fig. 1, we summarize our analysis strategy.

### Domain information analysis

Taxonomic domain information analysis was conducted at the MG-RAST (Meta Genome Rapid Annotation using Subsystem Technology, v3.1) server at the Argonne National Library (http://metagenomics.nmpdr.org). Species richness was computed as the antilog of the Shannon diversity (Meyer et al. 2008). A single similarity search at this server allows retrieving similarities to several databases, including NCBI-nr, KEGG, SEED, egg-NOG, COG, etc.

### Taxonomic classification

Taxonomic classification was conducted by BLASTN against SILVA SSUref and LSUref databases release 108 with an *e* value of 1e^−5^ (Urich et al. 2008) followed by the annotation of BLAST output files using MEGAN (Huson et al. 2007). This was performed by the lowest common ancestor algorithm that assigned rDNA or rRNA sequences to the lowest common ancestor in the taxonomy from a subset of the best scoring matches in the BLAST result (absolute cutoff BLAST bitscore 86, relative cutoff 10 % of the top hit) (Urich et al. 2008) using MEGAN according to these cutoffs to select hit reads for annotation. Randomly sequence reads exhibit very different levels of evolutionary conservation; therefore, it is important to make use of all ranks of the NCBI taxonomy, placing more conserved sequences higher up in the taxonomy (i.e., closer to the root) and more distinct sequences onto nodes that are more specific (i.e., closer to the leaves, which represent species and strains).

## Results

### Sequence analysis

**Fig. 2:**
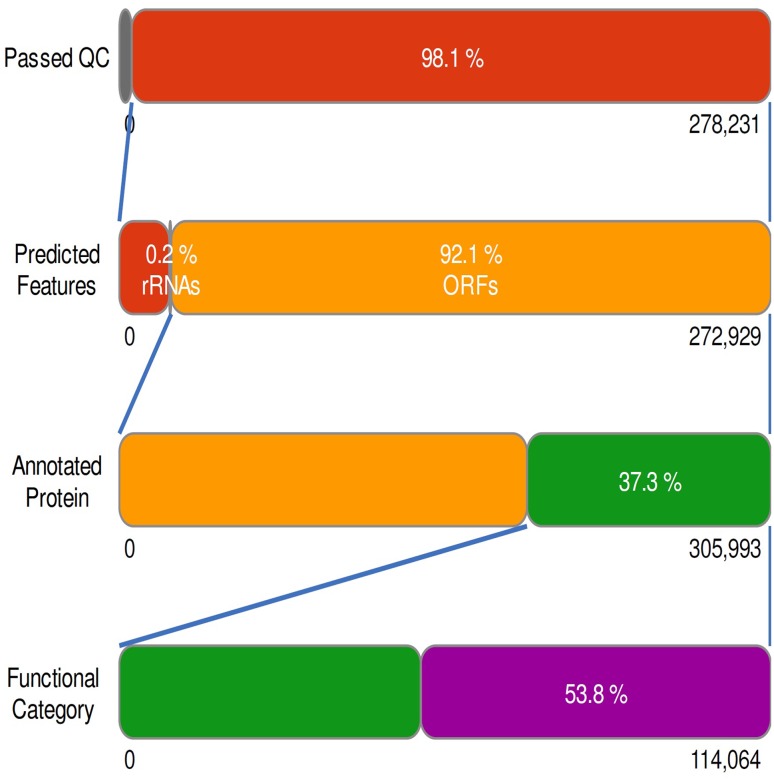
*Flow chart* depicting functional category hit distribution. 5302 sequences failed quality control. Of those, de-replication identified 1240 sequences (0.4 % of total) as artificial duplicate reads (ADRs). These include protein databases, protein databases with functional hierarchy information, and ribosomal RNA databases. The bars representing annotated reads are colored by *e* value range

Whole genome shotgun sequencing of the starter sample revealed 278,231 sequences containing 143,119,277 base pairs (bp) rendering an average read length of 641 bp. A total of 5302 sequences (1.9 %) failed to pass the quality-control (QC) pipeline. Of the sequences that passed QC, 590 sequences (0.2 %) contained ribosomal RNA genes. The remaining 119,194 sequences (42.8 %) contained predicted proteins with known functions and 132,223 sequences (47.5 %) contained predicted proteins with unknown function. About 20,917 (7.5 %) of the sequences that passed QC had no rRNA genes or predicted proteins. Out of the 272,929 sequences (totaling 143,119,277 bps) that passed quality control, 251,417 (92.1 %) produced 305,993 predicted protein coding regions. Of the 305,993 predicted protein features, a total of 114,064 (37.3 %) sequence reads had annotation with at least one of the protein databases (M5NR) and 191,929 (62.7 % of features) had no significant similarities to any protein databases, and about 61,377 features (53.8 % of annotated features) were assigned with functional categories, as described in the flow chart (Fig. 2; Table 2).

**Table 2:** Sequence statistics for the starter sample covering raw and high-quality sequence data used in the downstream analysis

Analysis statistics	Values (in number)
Number of contig	278,231
Total contig length	178,534,809
Maximum contig length	213,370
Minimum contig length	275
Base-pair count	178,540,333
Mean sequence length	641 ± 1650
Mean GC percent	42 ± 10
Artificial Duplicate Reads (ADRs)	1240
Post QC base-pair count	143,119,277
Post QC sequence count	272,929
Mean sequence length	524 ± 397
Predicted protein features	305,993
Predicted rRNA features	35,780
Identified protein features	114,064
Identified rRNA features	476
Identified functional categories	61,377

### Metagenome phylotyping (MG-RAST ID: 4556318.3)

**Fig. 3:**
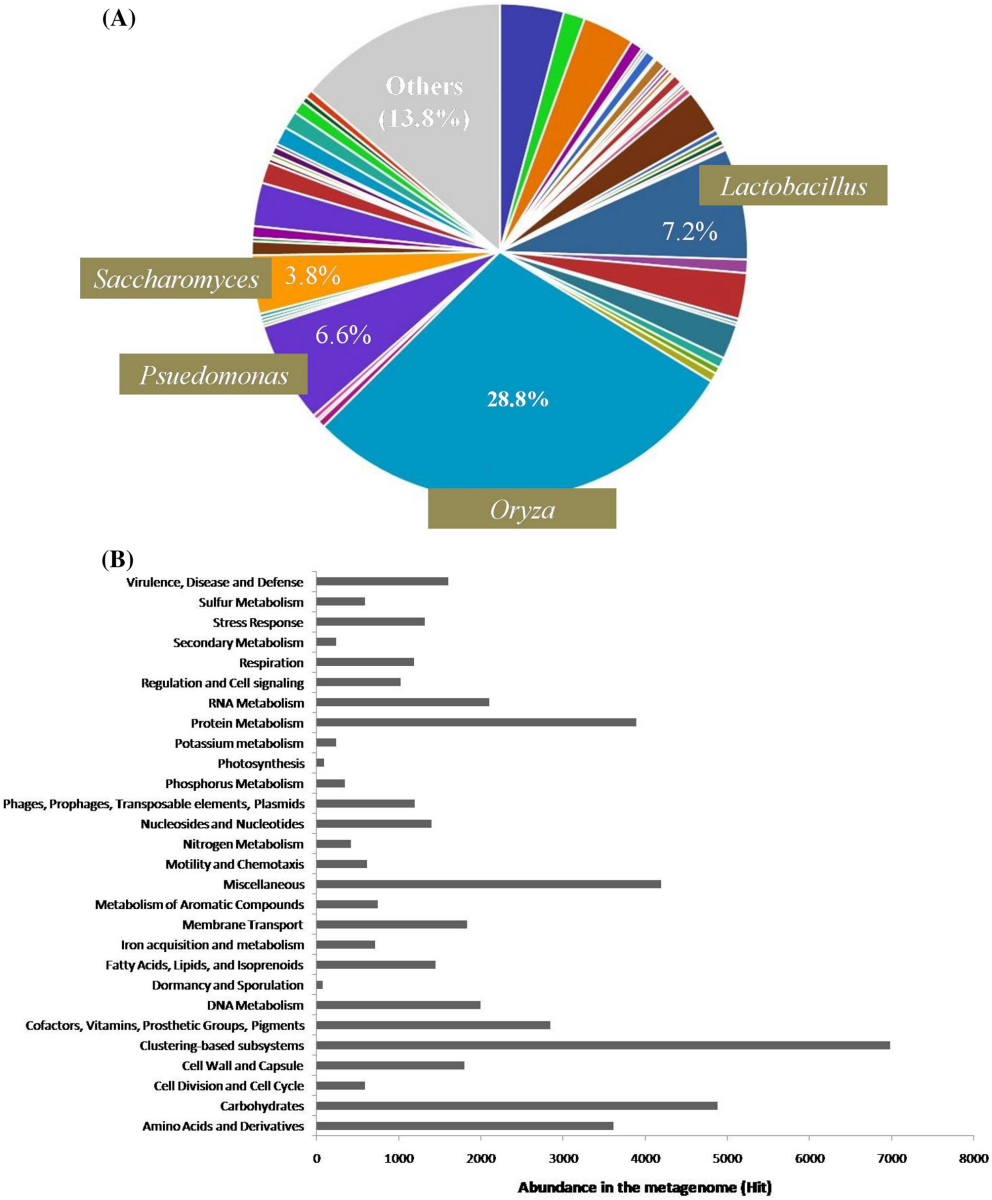
MG-RAST analyses of starter sample WGS metagenomics sequences. **a** Genus-level assignment of the sequences; **b** Functional annotation of predicted protein sequences

Cluster of Orthologous Groups (COGs) had 52,245 sequences predicted functions. Out of which, 21 % were identified with cellular processes and signaling, 21.7 % performed to information storage and processing, and 34.9 % could be attributed to metabolism functional categories. KEGG Orthology (KO) Functional Category had 31,626 sequences with predicted functions. Out of which, 46.9 % could be assigned to metabolism, 22.7 % to genetic information processing, 14.3 % to environmental information processing, and 11.8 % to cellular processes. Subsystem Functional Category had 47,970 sequences with predictable functions. 10.2 % of these sequences were associated with carbohydrates, 8.1 % with protein metabolism, and 7.5 % with amino acid derivatives functional categories (Fig. 3). Of the sequences that passed QC, 590 sequences (0.2 %) contained ribosomal RNA genes. MG-RAST analysis showed that at domain level, Eukaryota were the dominant domain, accounting for 60.4 % sequences, while bacteria comprised approximately 39.2 %. Sequences from viruses and others only accounted for 0.4 %. The genus-level assignments made by MG-RAST are shown in Fig. 3a. Among microbes, the genus *Lactobacillus* dominated the profile with 7.23 % total abundance, followed by the genre *Pseudomoonas* (6.59 %), *Acidovorax* (4.17 %), *Saccharomyces* (3.80 %), and *Leuconostoc* (2.98 %) with varying but comparable abundance.

**Fig. 4:**
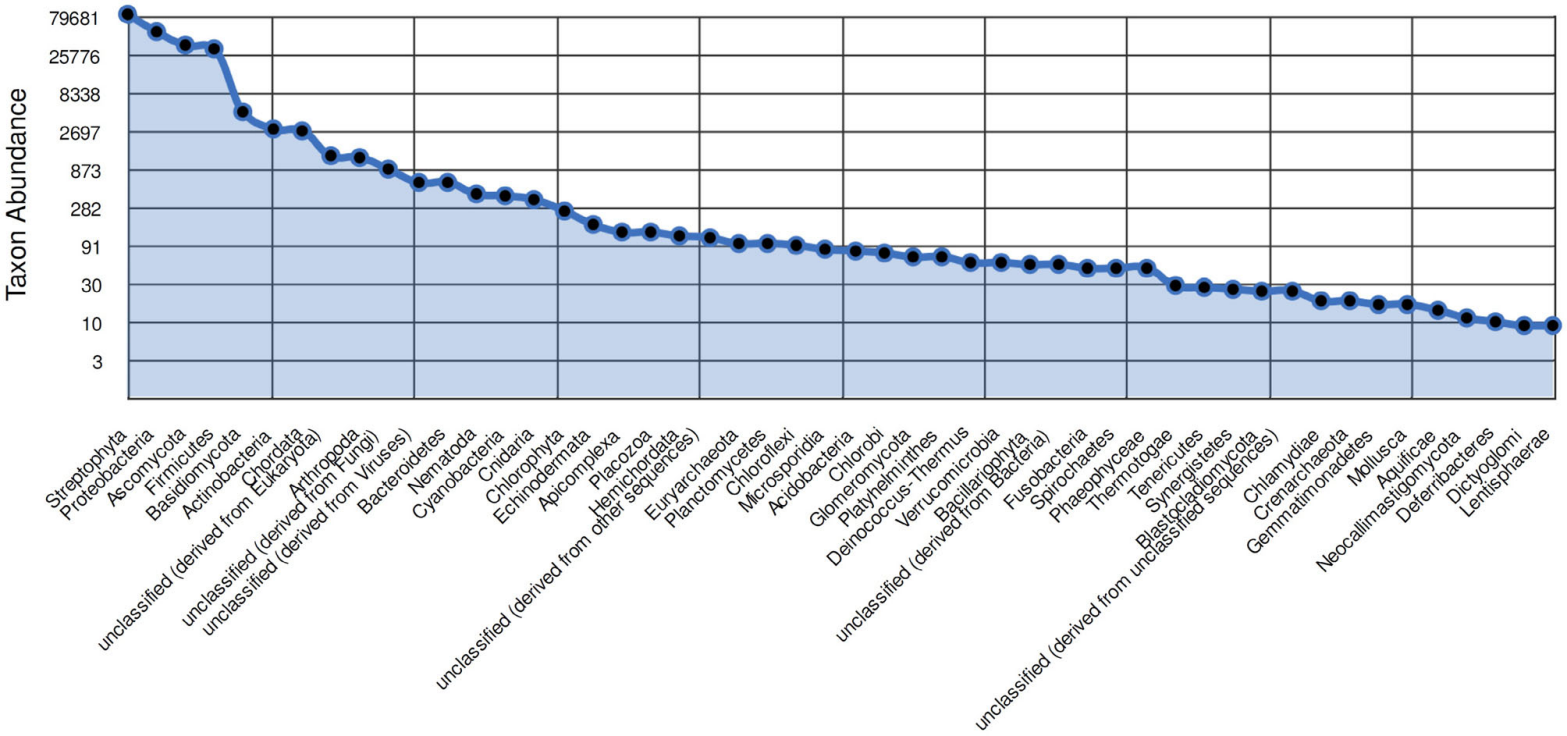
Phylum abundance ordered from the most abundant to least abundant. Only the top 50 most abundant are shown. The *Y-axis* plots the abundances of annotations in each phylum on a log *scale*. The rank abundance curve is a tool for visually representing taxonomic richness and evenness

At phylum level, a total of 38.7 % sequences belonged to streptophyta, 23 % sequences belonged to proteobacteria, and 15.8 % belonged to ascomycota. Taxonomic distribution at family level revealed that a total of 32.4 % sequences belonged to poaceae, 7.7 % belonged to lactobacillaceae, and 6.7 % sequences belonged to pseudomonadaceae (Fig. 4).

**Fig. 5:**
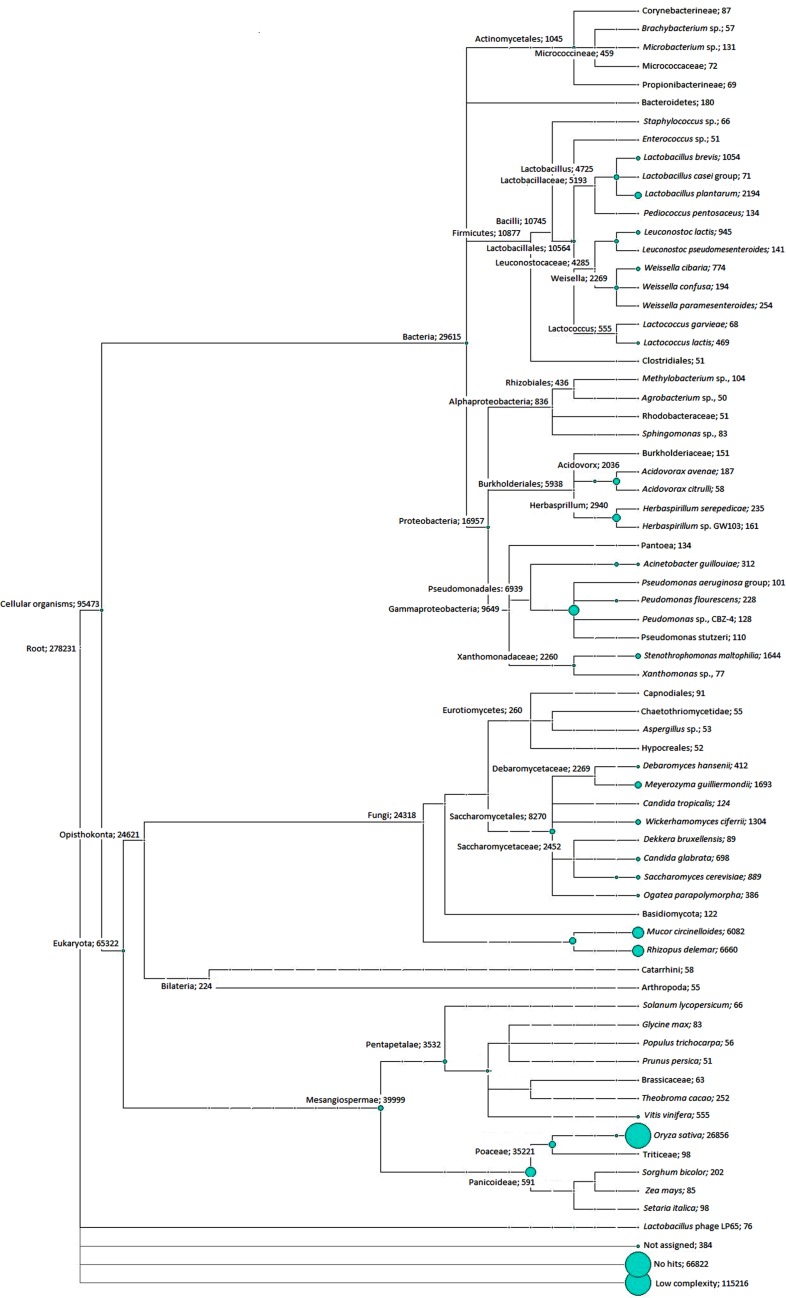
Phylogenetic diversity was computed using the LCA algorithm based on a BLASTX comparison of all the contigs against the NCBI-NR database. Each circle represents a taxon in the NCBI taxonomy and is labeled by its name and the number of contigs that are assigned either directly to the taxon, or indirectly via one of its subtaxa. The size of the circle is scaled logarithmically to represent the number of contigs assigned directly to the taxon

The assembled contigs were subjected to annotation using the LCA algorithm. The annotation was performed by aligning the contigs to non-redundant database of NCBI using BLASTX. These annotated files were imported to MEGAN v.5.2.3, and the downstream analysis was carried out. In Fig. 5, we show the LCA plot of these species based on the SSU rRNA analysis. Maximum contigs were assigned to *Oryza sativa* (26,856 contigs), followed by *Rhizopus delemar* (6660 contigs), *Mucor circinelloides* (6082 contigs), *Lactobacillus plantarum* (2194 contigs), *Meyerozyma guilliermondii* (1693 contigs), *Stenotrophomonas maltophilia* (1644 contigs), *Wickerhamomyces ciferrii* (1304 contigs), and *Lactobacillus brevis* (1054 contigs).

## Discussion

Rice wine is popular among most of the ethnic communities of Assam. They are prepared and consumed during various religious and harvest festivals. The preparation of rice wine involves the conversion of cooked rice by physical, microbiological, and biochemical operations, including steaming, inoculation with starter, and fermentation. A wide range of microorganisms are involved during fermentation processes, but only a few determines the quality of the endproduct. Three major microbial groups, namely molds, yeasts, and lactic acid bacteria, are reported to be involved in the traditional rice wine starters (Hesseltine et al. 1988; Steinkraus 1989; Lim 1991). Rice wine fermentation basically involves two major steps, viz., liquefaction and saccharification and alcoholic fermentation. In the first step, i.e., liquefaction and saccharification, fermentable sugars are produced from starch by the action of a-amylase and amyloglucosidase from molds which occurs as aerobic solid-state fermentation proceeds. Some yeast can also degrade starch, but this trait is not widespread (Laluce and Mattoon 1984; De Mot et al. 1985). The endproducts in this step are mainly glucose, and also to some extent dextrins and maltose (Crabb 1999). The second step of rice wine fermentation, i.e., alcoholic fermentation, involves conversion of fermentable sugars into alcohol by various yeasts. Some molds are also efficient in alcoholic fermentation. Different bacteria, mainly lactic acid bacteria (LAB), occur as opportunistic contaminants (Gandjar 1999; Thanh et al. 2008).

Controlled rice wine fermentation can be carried out using commercial or defined mixed starters which contain well-characterized efficient fungal and yeast strains (Dung et al. 2005). However, such products lose their traditional, characteristic taste, and odor. An ideal starter culture should enable obtaining fermented products with the palatability equal to good quality products, obtained as a result of spontaneous fermentation (Jazwiak et al. 2013).

### Microbial diversity and abundance

MG-RAST analysis of the metagenomic sequences revealed an alpha-diversity of 68.836 species. The rarefaction curve, which shows the observed operational taxonomic unit (OTU) richness as a function of the number of sequences sampled, indicated that the sequencing depth was insufficient to wholly capture the diversity present, indicating that the current sampling of the communities is insufficient and a large fraction of the species diversity remains to be discovered with more intensive sampling.

### Fungal diversity prevalent in *xaj*-*pitha*

Amylolytic fungi, such as *Mucor* sp., *Rhizopus* sp., *Amylomyces rouxii*, etc., have already been reported to be dominant in *bakhar*, a reported Indian starter culture (Tamang and Sarkar 1995). PCR-DGGE analysis revealed that four species of filamentous fungi belonging to the family Mucoraceae (*Rhizopus oryzae*, *Rhizopus microsporus*, *Absidia corymbifera*, and *Amylomyces* sp.) were dominant in traditional Vietnamese starter culture, “*banh men*” (Thanh et al. 2008). All these filamentous fungi are strong amylase producers and are frequently found in Asian fermentation starters (Hesseltine 1983; Hesseltine et al. 1988; Haard et al. 1999). Dominant fungal diversity that occurred in “*hong qu starter*” (starter for Chinese glutinous rice wine) is *R. oryzae, R. microspores, Mucor circinelloides, Penicillium chrysogenum, Aspergillus awamori, Aspergillus niger, Aspergillus flavus, Aspergillus oryzae, Monascus purpureus*, and *Monascus ruber* (Lv et al. 2013).

Three species of amylolytic filamentous fungi viz., *Rhizopus delemar*, *Mucor circinelloides* (Family: Mucoraceae), and *Aspergillus* sp. (Family: Trichocomaceae), were found in the present study of “*xaj*-*pitha*”. Mucor mycotina, including *Rhizopus*, *Amylomyces*, and *Mucor*, are indispensible microorganisms for the production of fermented foods in East Asia. Members of the genus *Rhizopus* specifically have often been reported from various fermented foods in eastern and southeastern Asia. Taxonomic contribution from Schipper and Stalpers (1984), Schipper (1984), Yuan and Jong (1984), Ellis (1985), Weitzman et al. (1996) etc., has consolidated the presence of 13 species in the group. *R. oryzae* is the most common fungus of any kind in the community prevalent in *banh men*, having been identified in 47 out of 52 tested samples. *Rhizopus* species, such as *R. oryzae* strains 28,168, 28,627, and 34,612, exhibited α-amylase activity (42.2, 76.0, and 40.4 U g^−1^, respectively) in solid-state fermentation (Soccol et al. 1994). During fermentation, this fungus binds dehulled and cooked substrate into a solid cake covered with a dense cottony mycelium.

*Mucor circinelloides* produces a complete set of cellulose degrading enzymes (Huang et al. 2014), indicating that this fungus could potentially be used in biomass conversion. However, *Mucor circinelloides* has also been identified as the causal agent of primary invasive cutaneous and maxillofacial zygomycosis (Iwen et al. 2007; Khan et al. 2009). Therefore, safety issues related to the presence of *Mucor circinelloides* in ethnic starters should be examined carefully.

### Yeast diversity prevalent in *xaj*-*pitha*

In a wine making industry, yeasts play the most significant role, as they are the key to alcoholic fermentation. Major yeast diversity found in *xaj*-*pitha* *were Meyerozyma guilliermondii, Wickerhamomyces ciferrii, Saccharomyces cerevisiae, Candida glabrata, Debaryomyces hansenii, Ogataea parapolymorpha*, and *Dekkera bruxellensis* (in descending order of their relative abundance). Previous studies have reported that *Saccharomycopsis* *fibuligera*, *S. cerevisiae*, *Wickerhamomyces* *anomala*, *Pichia guilliermondii*, and *Candida* sp. are the most common yeasts in rice wine starters (Lee and Fujio 1999; Xie et al. 2007; Jeyarama et al. 2008). A comprehensive assessment of yeast diversity occurred with “*hong qu starter*” (starter for Chinese glutinous rice wine) revealed the presence of *Saccharomyces cerevisiae, Saccharomycopsis fibuligera, Pichia fabianii, Candida glabrata, Cryptococcus heveanensis, Cryptococcus albidus, Saccharomycopsis malanga, Rhodotorula mucilaginosa, Sporobolomyces nylandii, Wickerhamomyces anomalus*, and *Rhodosporidium toruloides* (Lv et al. 2013). On the other hand, *Saccharomycopsis fibuligera, Saccharomyces cerevisiae, Issatchenkia* sp.*, Pichia anomala, Clavispora lusitaniae, Candida tropicalis*, and *Pichia ranongensis* were the yeast species found to occur in traditional Vietnamese alcohol fermentation starter “*banh men*” (Thanh et al. 2008). We assume that higher yeast diversity as reported in these studies could have resulted from a larger sampling population.

The yeast, *Saccharomycopsis* (*Endomycopsis*) *fibuligera*, possessing amylolytic and ethanol producing capacity, is one of the common yeasts present in traditional rice wine starter (Limtong et al. 2002). In our study, *Saccharomycopsis* *fibuligera* could not be detected in the starter. However, the predominant yeasts in alcoholic fermentation belong to the genus *Saccharomyces*, especially *S. cerevisiae* (Battcock and Ali 1993). It is the most effective ethanol producer known so far (Vaughan-Martini and Martini 1995). *Meyerozyma guilliermondii* (previously named *Pichia guilliermondii*) has been found to be effective towards different postharvest spoilage fungi, such as *Penicillium digitatum* on grapefruit (Droby et al. 1989), *Botrytis cinerea* and *Penicillium expansum* on apples (Wisniewski et al. 1991), and *Aspergillus flavus* on soybeans (Paster et al. 1993). The yeast may act as a biocontrol agent against spoilage microorganisms increasing the shelf life of the product. The antagonistic effect shown by the yeast against *P. digitatum* and *B. cinerea* is mainly due to the competition for nutrients and the secretion of cell wall degrading enzymes (Petersson and Schnürer 1995).

*Debaryomyces hansenii* is extremophilic yeast; it has been isolated from food products, such as cheese, meat, wine, beer, fruit, etc. (Norkrans 1966; Davenport 1980; Barnett et al. 2000) as well as from some other high-sugar products (Tilbury 1980). This high lipid accumulating, osmotolerant (Onishi 1963) yeast has shown some interesting genetical and biochemical features for upcoming biotechnological applications (Baronian 2004; Ratledge and Tan 1990). Unfortunately, *D. hansenii* is also well known for spoilage of brine-preserved foods, such as gherkins (Breuer and Harms 2006).

A wide variety of spoilage yeasts, e.g., *Pichia* sp., *Zygosaccharomyces* sp., *Kluyveromyces* sp., *Brettanomyces* sp., etc., have also been reported from alcoholic fermentation, which may spoil wines during storage through changes of biochemical activities (Fleet 1993). Out of these, members of the genus *Brettanomyces* (imperfect state, *Dekkera*) are probably the most serious and controversial spoilage yeasts (Cocolin et al. 2004). These yeasts have been reported from almost every wine-producing area of the world (Fungelsang 1997). They have also been isolated from other fermented beverages, such as beer and cider. Osmo-/alcohol-tolerant *Brettanomyces* species can survive and contaminate wines in an ill-managed setup. *Brettanomyces*/*Dekkera*-contaminated wine develops off-flavors and a distinct haziness, which severely decreases product market value (Chatonnet et al. 1992, 1995; Edlin et al. 1995).

### Lactic acid bacteria (LAB) prevalent in *xaj*-*pitha*

The group of Lactic acid bacteria (LAB) was found to be the most diverse bacterial as well microbial group with 4 genera and 12 species detected. The following species were found to be most abundant: *Lactobacillus plantarum*, *Lactobacillus brevis*, *Leuconostoc lactis*, *Weissella cibaria*, *Lactococcus lactis*, *Weissella para mesenteroides*, *Weissella confusa*, *Leuconostoc pseudomesenteroides*, *Pediococcus pentosus*, *Lactobacillus casei* group, *Lactococcus garvieae*, and *Enterococcus* sp. Major LAB diversity viz, *Weissella paramesenteroides, Pediococcus pentosaceus*, and *Pediococcus acidilactici* have been reported to occur in *“hong qu* starter*”* (a Chinese glutinous rice wine) (Lv et al. 2013). On the other hand, *Enterococcus faecium, Lactobacillus agilis, Lactobacillus brevis, Lactobacillus fermentum, Lactobacillus manihotivorans, Lactobacillus plantarum, Lactobacillus* sp., *Lactococcus lactis, Leuconostoc citreum, Leuconostoc garlicum, Leuconostoc mesenteroides, Pediococcus pentosaceus, Weissella confusa*, and *Weissella paramesenteroides* have been reported from traditional Vietnamese alcohol fermentation starter *Banh men* (Thanh et al. 2008).

Acidification caused by this group of bacteria favors the growth of amylolytic yeast and fungi while suppressing the growth of unwanted spoilage and pathogenic bacteria (Gandjar 1999; Haard et al. 1999). Probably abundance of LAB is also the reason behind most of the off-flavored and hazy colored rice wine with low shelf life. Many of these *Lactobacillus* strains are already in the pharmaceutical industry as probiotics agents; however, their credibility as good probiotics for human usage is still under debate and needs further clinically supporting data (Reid 1999).

### Plant pathogens/environmental contaminants

The major plant pathogens viz*., Acidovorax avenae*, *Acidovorax avenae* subsp. *avenae* (*Pseudomonas avenae*), *Acidovorax avenae* subsp. *citrulli*, *Herbaspirillum seropedicae*, *Herbaspirillum* sp. GW103, *Pantoea*, *Methylobacterium*, *Sphingomonas*, *Xanthomonas*, etc., were detected in the sample. Some other environmental (from soil and water) contaminants, such as *Pseudomonas fluorescens*, *Pseudomonas* sp. CBZ-4, *Pseudomonas stutzeri*, *Psuedomonas aeruginosa* group, *Stenotrophomonas maltophilia*, etc., were also detected. Ethnic fermentation process is carried out at rural household level, where hygiene is often compromised. Several opportunistic human skin commensals, such as *Acinetobacter guillouiae,* *Microbacterium* sp., *Micrococcus* sp., *Staphylococcus* sp., etc., were detected. Out of these, *Acinetobacter guillouiae* has received increasing attention during the recent times as significant opportunistic pathogen, usually in the context of serious underlying disease in immune-compromised patients from south-east Asia and tropical Australia (Dijkshoorn et al. 2007, Peleg et al. 2008). We presume that all contaminants (plant pathogen/environmental contaminant/clinical contaminant) in the starter were introduced along with the plant parts, or through unhygienic practices.

### Herbs

It has been observed that different rice wine-producing communities follow almost identical protocols for fermentation, but they use different species of plants in the starter culture preparation, which are believed to add as an intoxicating property to the liquor (Sarma 2002). Ethnic communities of North East India have been regularly using several herbs and plants, such as *Albizia myriophylla*, *Amomum aromaticum*, *Plumbago zeylanica*, *Buddleja asiatica*, *Vernonia cinerea*, *Gingiber officinale*, *Glycyrrhiza glabra*, *Ananas comosus*, *Artocarpus heterophyllus*, *Calotropis gigantea*, *Capsicum frutescens*, etc. (Das et al. 2012). In South Asian country Cambodia, spice plants, such as *Capsicum* sp., *Alpinia* sp., *Piper* sp., *Allium sativum*, *Zingiber officinale*, *Illicium verum*, *Amomum krervanh*, *Cinnamomum* sp., etc., and sweet ingredients (*Albizia* sp., *Cinnamomum* sp., *Saccharum officinarum*) are commonly used (Das et al. 2012; Seesuriyachan 2011). Apart from imparting color, flavor, and sweetness to the wine, the various plants used in the starter culture are also said to have many medicinal properties. Some of the plant extracts may also provide certain nutrients for the survival of the microflora present in the starter cakes. The effects of the herbs used in traditional starter preparations on the starter microflora have been studied (Phuc 1998; Dung et al. 2005). It was suggested that some herbs have a stimulatory effect on biomass and also on yeasts count. Particularly, the herbs “Tieu Hoi” (Fennel: *Foeniculum vulgare* Miller) and “Dinh Huong” (Clove: *Syzygium aromaticum* L.) used in Vietnamese starter culture prove to be stimulatory in biomass production of molds and yeasts (Dung et al. 2005). Our metagenomics study has revealed the presence of several plant parts viz., *Prunus persica*, *Setaria italica*, *Glycine max*, *Solanum lycopersicum*, *Brassica* sp., *Vitis vinifera*, etc., in the starter. However, any stimulatory or inhibitory effects of these plant parts are yet to be tested. Nonetheless, Ppdfn1, a defensin protein, from *Prunus persica* (common name peach) shows antifungal activity against pathogens, such as *Botrytis cinerea*, *Monilinia laxa*, and *Penicillium expansum* (Nanni et al. 2013). Interestingly, phyllosphere of *Setaria italica* has been reported to harbor some of the dominant fungi, such as *Rhizopus nigricans*, *Curvularia pallescens*, *Aspergillus flavus*, *A. fumigatus*, and *Trichoderma album* (Upadhyaya and Gupta 2009). This indicates that the herbs and other plants may also be a source of some of the microbial mass which could play an important role in fermentation. At this point, we assume that a study on microbial dynamics, including succession and niche differentiation, during the actual alcohol fermentation could be an interesting topic for further research.

## Conclusion

The MG-RAST analysis showed that the analyzed starter sample had an alpha-diversity of 68.84 species. Taxonomic hit distribution of the sample at domain level showed that 39.2 % sequences belonged to Bacteria and 60.4 % sequences belonged to Eukaryota. Taxonomic hits of distribution at phylum level showed that 38.7 % sequences belonged to Streptophyta, 23 % sequences belonged to Proteobacteria, and 15.8 % belonged to Ascomycota. According to the MEGAN analysis, the total 2,78,231 sequences were found to have maximum abundance of *Rhizopus delemar* followed by *Mucor circinelloides*, *Lactobacillus plantarum*, *Meyerozyma gulliermondii*, and so on. Along with some efficient molds and yeasts, a wide range of environmental (opportunistic) contaminants were also found, which may pose serious health hazards. At this point, we assume that the main difference between traditional and industrial starters is that traditional starters have a higher resilience over an industrial one. Therefore, it may be suggested that a study on changes of microbiota during spontaneous fermentation at different time points would throw more light into the role of the various microorganisms.
